# Stable surface display of a conserved influenza mini-HA antigen in *Saccharomyces cerevisiae* for oral delivery

**DOI:** 10.1186/s13568-026-02101-w

**Published:** 2026-08-16

**Authors:** Yu Zhang, Xiaoying Zhi, Zeliang Guo, Wenqi Xu, Bingyu Li, Rongzeng Liu

**Affiliations:** 1 Department of Immunology, College of Basic Medicine and Forensic Medicine, Henan University of Science and Technology, Luoyang, China; 2 School of Medicine, Zhoukou Polytechnic, Zhoukou, China; 3 Key Laboratory of Clinical Immunology and Inflammatory Diseases, Luoyang, China

**Keywords:** Yeast surface display, *Saccharomyces cerevisiae*, Recombinant protein presentation, Antigen stability, Thermal stability, Oral delivery

## Abstract

Yeast surface display is a versatile microbial engineering strategy for recombinant protein presentation and oral antigen delivery. In this study, a conserved influenza hemagglutinin stem antigen (mini-HA) was displayed on the surface of *Saccharomyces cerevisiae* to evaluate the feasibility and stability of a yeast-based oral delivery system. A rationally engineered mini-HA derived from influenza A H1N1 was anchored to the yeast cell surface using the Aga1p-Aga2p display system. Efficient surface localization was confirmed by flow cytometry and confocal microscopy. The displayed antigen remained stably anchored following exposure to simulated gastric fluid and after heat inactivation at 60 °C, indicating favorable stability of the yeast surface display system under acidic and thermal stress conditions. Oral administration of the engineered yeast induced antigen-specific systemic IgG and mucosal secretory IgA responses in mice. In addition, antigen-specific lymphocyte proliferation and increased expression of IFN-γ and T-bet were observed, suggesting activation of both humoral and cellular immune responses. No significant adverse effects on body weight, serum biochemical parameters, or tissue histopathology were detected. These findings support the feasibility of using *S. cerevisiae* surface display for stable presentation and oral delivery of a conserved influenza antigen. The favorable stability and immunogenicity of the system support further development of yeast-based recombinant antigen delivery platforms.

**Supplementary Information:**

The online version contains supplementary material available at https://doi.org/10.1186/s13568-026-02101-w.

## Introduction

Respiratory viral infections continue to pose a substantial global health burden, underscoring the urgent need for vaccine strategies capable of inducing durable protection at mucosal surfaces. Influenza A (H1N1), a highly transmissible respiratory virus, exemplifies this challenge due to its continuous antigenic drift and capacity to trigger both seasonal epidemics and global pandemics, such as the 2009 H1N1 outbreak (Li et al. 2022; Xing et al. 2021; Xu et al. 2020). Antigenic variation in viral surface glycoproteins frequently undermines pre-existing immunity, necessitating the recurrent reformulation of seasonal influenza vaccines and highlighting limitations in current vaccination strategies.

Hemagglutinin (HA), a trimeric glycoprotein displayed on the viral envelope, represents the primary antigenic component of influenza vaccines and mediates viral attachment and membrane fusion with host cells (Jiao et al. 2023). Structurally, HA consists of a globular head domain (predominantly HA1) and a stem domain (predominantly HA2) (Zhang et al. 2024). While the immunodominant head domain exhibits substantial variability that drives antigenic drift and strain-specific immune escape, the stem domain is highly conserved across influenza A subtypes and has been shown to elicit broadly neutralizing antibodies (Crofts et al. 2025; Wu and Wilson 2020). Consequently, the HA stem has emerged as a promising target for the development of broadly protective or “universal” influenza vaccines.

Despite advances in antigen design, the successful translation of conserved viral antigens into effective vaccines critically depends on the availability of delivery platforms that can preserve antigen conformation, enhance immunogenicity, and promote protective immunity at mucosal sites. Yeast surface display technology offers a unique combination of eukaryotic protein expression and intrinsic immunostimulatory properties, making it an attractive platform for vaccine development (Kumar and Kumar 2019). The eukaryotic expression machinery of *Saccharomyces cerevisiae* (*S. cerevisiae*) facilitates accurate folding and presentation of complex viral antigens, thereby preserving their native conformation and immunogenic epitopes, including those found in trimeric HA structures (Byrd-Leotis et al. 2022). In addition, β-1,3-glucans within the yeast cell wall function as natural adjuvants by engaging pattern recognition receptors (PRRs) on antigen-presenting cells, leading to enhanced antigen uptake and immune activation (He et al. 2024).

Importantly, yeast-based vaccine platforms are particularly well suited for oral administration. The acid-resistant yeast cell wall confers protection against degradation in the gastrointestinal tract, enabling efficient delivery of surface-displayed antigens to gut-associated lymphoid tissues. Oral administration of whole yeast-displayed vaccines has been shown to induce coordinated systemic and mucosal immune responses, characterized by robust secretory IgA (sIgA) production and the generation of tissue-resident memory T cells, both of which are essential for long-term mucosal immunity (Silva et al. 2023). Moreover, proteins displayed on the yeast surface exhibit remarkable physicochemical stability, maintaining structural integrity for extended periods under both refrigerated and ambient storage conditions, which is advantageous for vaccine distribution and deployment(Kumar 2018). From a safety and translational perspective, *S. cerevisiae* is approved by the U.S. Food and Drug Administration and is classified as generally recognized as safe (GRAS), further supporting its suitability as a vaccine delivery vehicle (Vanderwaeren et al. 2022).

In this study, we engineered an orally deliverable vaccine platform based on *S. cerevisiae* surface display of a conserved H1N1 HA stem antigen (mini-HA). By genetically fusing mini-HA to the cell wall anchor protein Aga2p, the antigen was covalently attached to Aga1p on the yeast surface, enabling direct oral delivery without the need for complex protein purification. Using the influenza A virus as a representative model antigen, we observed that oral immunization with heat-inactivated yeast-displayed mini-HA (YSD-mini-HA) induces robust antigen-specific immune responses in mice, characterized by a pronounced Th1-biased cellular response and strong mucosal sIgA production. Together, these findings suggest a genetically modular, stable, and cost-effective yeast-based oral vaccine platform with potential applicability to influenza and other emerging respiratory pathogens.

## Materials and methods

### Construction of yeast surface display plasmid

**Fig. 1:**
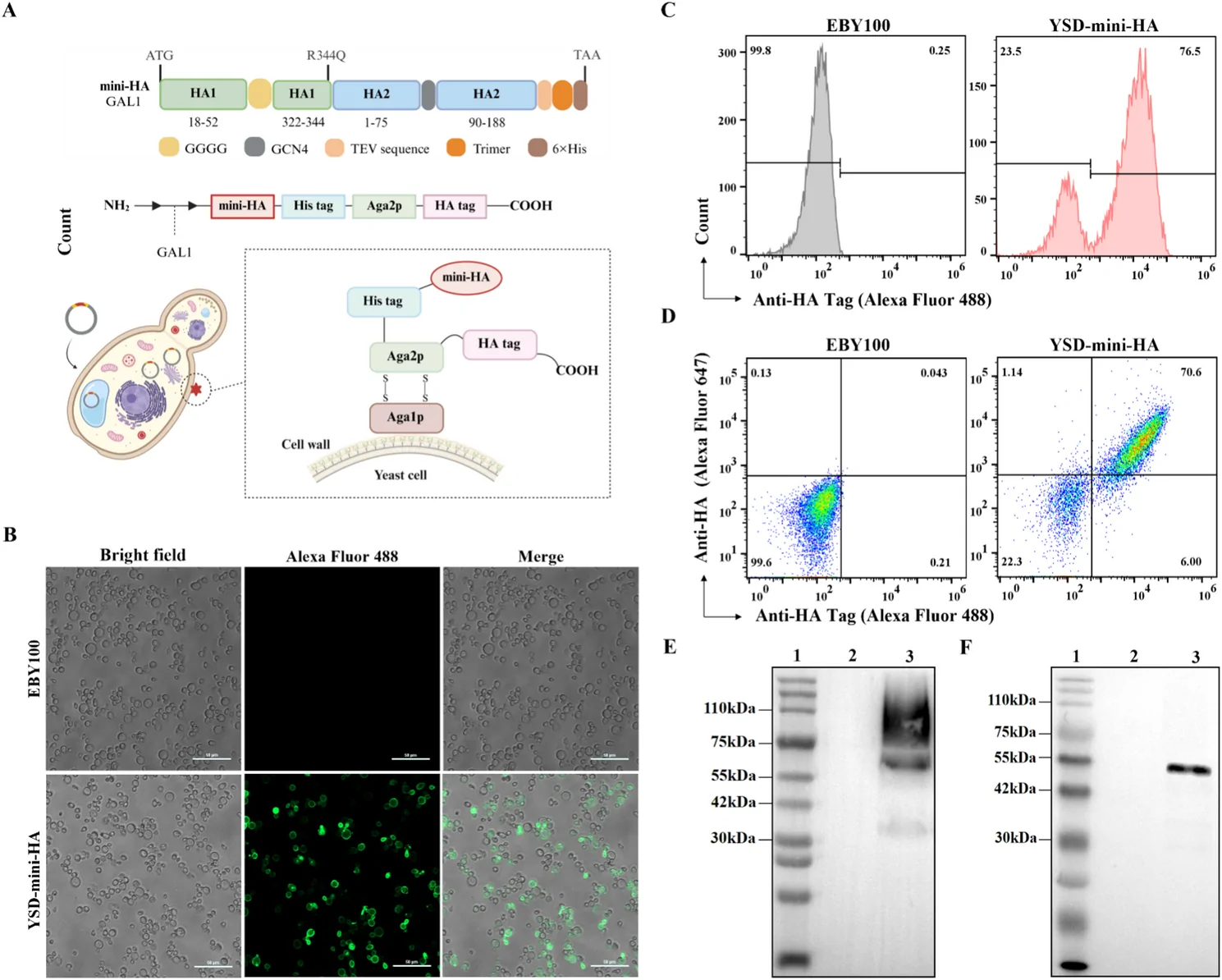
Surface display of a mini-HA on *S. cerevisiae*. **A** Schematic illustration of the yeast surface display system used for oral vaccine delivery. The mini-HA antigen was genetically fused to Aga2p and anchored to the yeast cell wall through interaction with Aga1p via the a-agglutinin system, enabling covalent attachment to β-1,6-glucan. **B** Representative confocal microscopy images showing surface expression of mini-HA on induced yeast cells. Green fluorescence indicates mini-HA expression. Scale bar, 50 µm. **C** Flow cytometric analysis of yeast cells labeled with anti-HA tag primary antibody followed by Alexa Fluor 488-conjugated secondary antibody. **D** Dual-label flow cytometric analysis using antibodies against the HA tag (Alexa Fluor 488) and mini-HA (Alexa Fluor 647), demonstrating co-expression on the yeast surface. Data are representative of at least three independent experiments. **E** mini-HA was analyzed by Western blot using an anti-HA-specific antibody. **F** PNGase F-deglycosylated mini-HA was analyzed by Western blot using an anti-HA-specific antibody. Experiments were repeated three times independently with similar results

The yeast shuttle vector used for surface display was derived from the previously described plasmid ptFLU (LEU⁺) (Jiang and Boder 2010), and the resultant recombinant plasmid was designated ptmini-HA. The mini-HA antigen was designed based on the hemagglutinin (HA) structure of influenza A/California/04/2009 (H1N1) (PDB: 3LZG), in which the globular head domain was removed and replaced by a flexible linker to generate a stabilized stem-only construct. To promote trimerization and enhance structural stability of the HA stem, a GCN4-derived trimerization motif was introduced. In addition, the HA proteolytic cleavage site was removed to preserve the prefusion conformation of the antigen. The sequences of mini-HA construct are shown in Supplementary Data S1. The conserved hemagglutinin stem antigen (mini-HA) derived from influenza A (H1N1) virus was genetically fused to the *S. cerevisiae* cell wall anchor protein Aga2p, under the control of the GAL1 promoter. An HA epitope tag (YPYDVPDYA), distinct from the influenza HA sequence, was incorporated to facilitate independent detection of surface expression (Fig. 1A). All constructs were confirmed by DNA sequencing prior to transformation.

### Yeast strain, transformation, and induction of surface expression

The *S. cerevisiae* strain EBY100 (URA^+^ and TRP^−^) was used for yeast surface display. Yeast transformation was performed using the lithium acetate method according to standard protocols. Transformed yeast cells were selected on SD-CAA plates containing 2% (w/v) dextrose (glucose), 0.67% (w/v) yeast nitrogen base without amino acids, 0.062% (w/v) Ura/Leu dropout casamino acids, 38 mM Na_2_HPO_4_, 62 mM NaH_2_PO_4_·H_2_O, and 1.5% agar (w/v). A single colony from the plates was subsequently inoculated into 2 mL of SD-CAA liquid minimal medium at 30 °C with shaking. For induction of surface expression, yeast cells were transferred to SG-CAA medium containing galactose and incubated at 30 °C for 24 h unless otherwise specified (Liu et al. 2022).

### Flow cytometry analysis of surface-displayed mini-HA

As previously detailed (Liu et al. 2021), induced yeast cells were washed with phosphate-buffered saline (PBS) and blocked with 1% bovine serum albumin (BSA). Surface-displayed mini-HA was detected using either mouse anti-HA tag antibodies (diluted 1:100; Merck KGaA, Darmstadt, Germany) or anti-HA antibodies (diluted 1:100; Sino Biological, Beijing, China), followed by fluorophore-conjugated secondary antibodies (diluted 1:100; Thermo Fisher Scientific, Waltham, MA, USA). For dual-labeling experiments, yeast cells were incubated sequentially with both primary antibodies. Flow cytometry was performed using a BD flow cytometer, and data were analyzed using FlowJo software. Briefly, cells were initially gated on FSC-A/SSC-A to exclude debris, followed by doublet exclusion using FSC-A/FSC-H. Fluorescence thresholds were set using untransformed EBY100 cells as the negative control, and the gated population was then analyzed for AF488 (anti-HA Tag Ab) and AF647 (anti-mini-HA Ab) fluorescence intensity. Mean fluorescence intensity (MFI) and the proportion of positive populations were quantified relative to isotype-matched antibody controls.

### Confocal microscopy

For confocal microscopy analysis, induced yeast cells were stained with the indicated primary and secondary antibodies as described above and mounted on glass slides. Images were acquired using a laser scanning confocal microscope. Fluorescence localization was assessed to confirm surface display of mini-HA.

### Western blot analysis

YSD-mini-HA cultures induced with galactose were harvested at OD_600_ = 1.0, washed with PBS, and lysed by boiling at 95 °C for 5 min. Protein extracts were deglycosylated with PNGase F (Beyotime, Shanghai, China), and 10 μL of each sample were resolved by SDS-PAGE and transferred to PVDF membranes. After blocking with 5% skim milk in TBST for 2 h at room temperature, membranes were incubated overnight at 4 °C with anti-HA primary antibody (1:2000, Sino Biological, Beijing, China), followed by three TBST washes and a 1 h incubation with HRP-conjugated secondary antibody (1:5000, Thermo Fisher Scientific, Waltham, MA, USA) at room temperature. Immunoreactive bands were visualized by ECL (Tanon, Shanghai, China) after three final washes. S. cerevisiae EBY100 served as the negative control.

### Stability analysis under simulated gastric conditions

To evaluate antigen stability under gastric conditions, induced yeast cells were incubated in simulated gastric fluid (SGF; pH 1.2) (Biosharp, Anhui, China) containing 3.2 mg/mL pepsin at 37 °C for up to 120 min. At indicated time points, samples were centrifuged and supernatants were collected for protein quantification using a BCA (Biosharp, Anhui, China) assay. Residual surface display of mini-HA on yeast cells was assessed by flow cytometry and confocal microscopy as described above.

### Thermal inactivation and surface display analysis

To prepare a non-replicative whole-yeast oral vaccine, induced YSD-mini-HA yeast cells were subjected to heat inactivation. Yeast suspensions were incubated at designated temperatures for 1 h. Following heat treatment, cells were washed with PBS and analyzed for surface retention of mini-HA by flow cytometry using anti-HA tag (Merck KGaA, Darmstadt, Germany) and anti-HA antibodies. Heat treatment at 60 °C was selected for subsequent immunization studies based on surface display stability.

### Animals and immunization protocol

Female BALB/c mice (6–8 weeks old) were randomly assigned to experimental groups. Mice were orally immunized with heat-inactivated YSD-mini-HA yeast (150 OD_600_ per dose). Control groups received heat-inactivated EBY100 yeast or PBS. Immunizations were administered according to the schedule illustrated in Fig. 4A. Serum and fecal samples were collected at indicated time points for immunological analyses.

### Measurement of antigen-specific antibody responses

Serum HA-specific IgG, IgG1, and IgG2a levels were measured by enzyme-linked immunosorbent assay (ELISA). Briefly, plates were coated with purified HA antigen (GenScript, Nanjing, China), blocked, and incubated with serial dilutions of mouse sera. Bound antibodies were detected using HRP-conjugated secondary antibodies specific for total IgG (Thermo Fisher Scientific, Waltham, MA, USA), IgG1, or IgG2a (Proteintech, Wuhan, China) and developed with TMB substrate (Solarbio, Beijing, China). Fecal samples were processed for the measurement of HA-specific secretory IgA (sIgA; Immunoway, Plano, TX, USA) using a similar ELISA protocol. Antibody titers were calculated as the reciprocal of the highest dilution exceeding the defined cutoff value.

### Analysis of cellular immune responses

Seven days after the final immunization, spleens were harvested and homogenized. Total RNA was extracted from splenic tissues and reverse-transcribed into cDNA (Takara, Beijing, China). Quantitative real-time PCR (RT-qPCR) was performed to assess the expression of cytokines and transcription factors associated with Th1 and Th2 responses (Table S1). Gene expression levels were normalized to housekeeping genes and calculated using the 2^−ΔΔCt^ method.

For lymphocyte proliferation assays, splenocytes were isolated and cultured in vitro in the presence of HA antigen (GenScript, Nanjing, China). Cell proliferation was assessed using a CCK-8 assay (Solarbio, Beijing, China) according to the manufacturer’s instructions. Concanavalin A (ConA; Solarbio, Beijing, China) was used as a positive control.

### Safety evaluation

Mice were monitored daily for clinical signs, behavior, and body weight throughout the immunization period. Serum alanine aminotransferase (ALT) and creatinine (CREA; Jiancheng, Nanjing, China) levels were measured using commercial biochemical kits. Major organs, including the heart, liver, spleen, lung, kidney and gastrointestinal tract were collected after the final immunization, fixed in formalin, embedded in paraffin, sectioned, and stained with hematoxylin and eosin (H&E) for histopathological examination.

### Statistical analyses

Data are presented as mean ± standard error (SE). Statistical analyses were performed using GraphPad Prism software. Differences between groups were analyzed using one-way or two-way ANOVA where appropriate. A *p* value < 0.05 was considered statistically significant.

## Results

### Efficient surface display of mini-HA on S. cerevisiae

To generate a yeast-displayed oral vaccine candidate, the ptmini-HA plasmid was transformed into the parental *S. cerevisiae* strain EBY100. The mini-HA antigen was genetically fused to Aga2p and anchored to the yeast cell wall via the a-agglutinin system, enabling surface presentation of the antigen. An HA epitope tag (YPYDVPDYA), distinct from the mini-HA sequence, was incorporated to facilitate independent detection of antigen expression (Fig. 1A).

Surface localization of mini-HA was first examined by confocal microscopy following galactose induction. Yeast cells expressing YSD-mini-HA exhibited strong, continuous fluorescence distributed along the cell periphery, forming a characteristic ring-like pattern, whereas no specific fluorescence was detected in the parental EBY100 strain (Fig. 1B). Flow cytometric analysis further demonstrated that 76.5% of induced yeast cells were positive for HA-tag staining, indicating efficient surface expression (Fig. 1C). Dual-labeling with antibodies specific for both the HA tag and mini-HA revealed that 70.6% of cells were double-positive, confirming the successful and specific display of mini-HA on the yeast surface (Fig. 1D). Western blot analysis confirmed the expression and glycosylation of mini-HA. As shown in Fig. 1E, no band was detected in the negative control EBY100 (lane 2). The YSD-mini-HA protein migrated at ~ 65 kDa (Fig. 1E, lane 3), which is much higher than its theoretical molecular weight (~ 48.4 kDa). Following PNGase F treatment, the band shifted to ~ 48.4 kDa (Fig. 1F, lane 3), confirming N-glycosylation.

### Stability of surface-displayed mini-HA under simulated gastric conditions

**Fig. 2:**
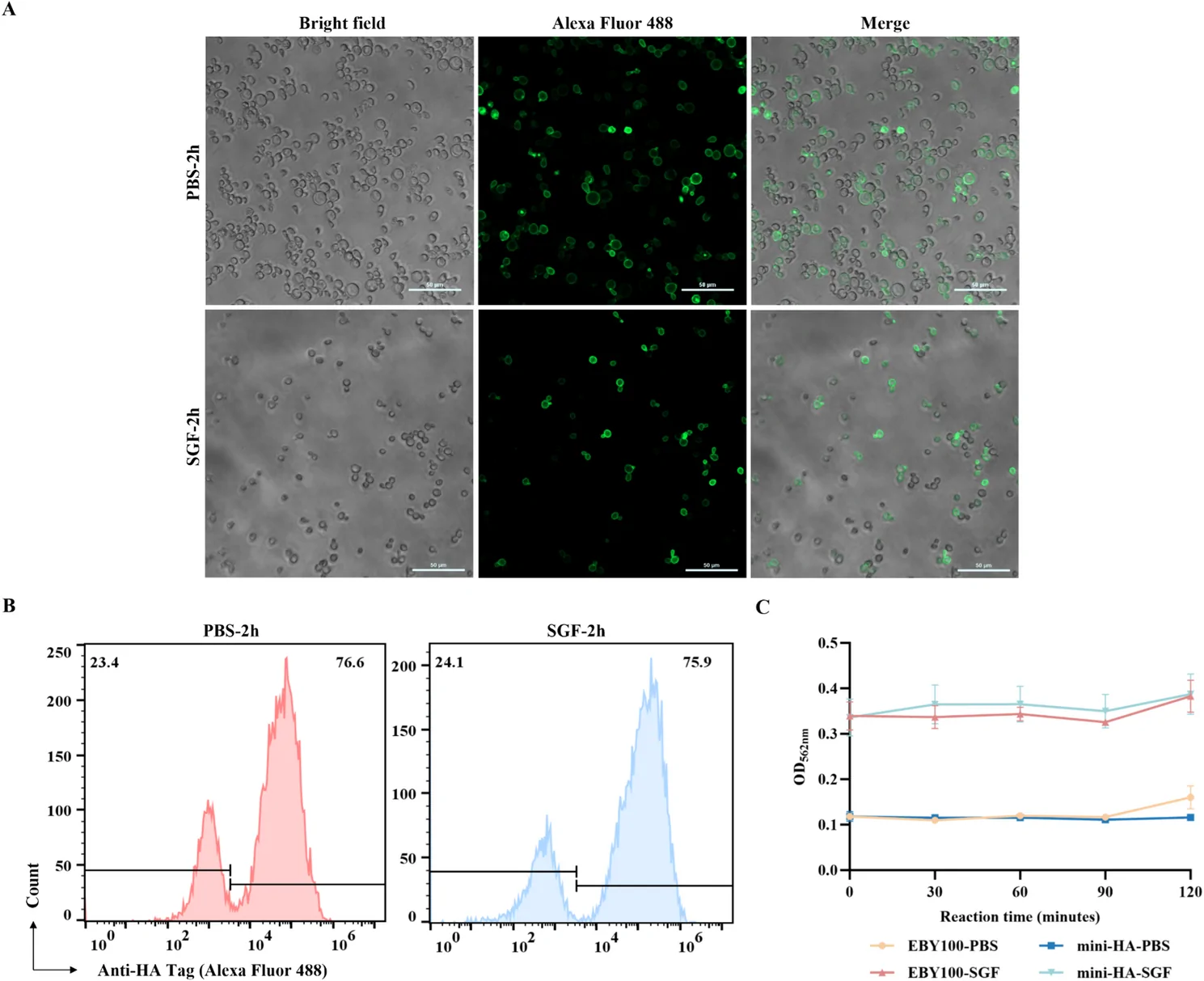
In vitro stability of yeast-displayed mini-HA under simulated gastric conditions. **A** Representative confocal microscopy images showing the retention and surface localization of yeast-displayed mini-HA after 2 h exposure to SGF. **B** Flow cytometric assessment of surface-displayed mini-HA on *S. cerevisiae* after 2 h exposure to SGF, indicating retention of surface-anchored antigen. **C** Quantification of total protein released from yeast cells following incubation in SGF for 0, 30, 60, 90, and 120 min, as measured by BCA assay. Experiments were repeated three times independently with similar results

To evaluate the suitability of YSD-mini-HA for oral administration, the stability of the surface-displayed antigen was assessed using an in vitro SGF model (pH 1.2, 3.2 mg/mL pepsin). Yeast cells were incubated in SGF for up to 120 min, and protein release into the supernatant was quantified at defined time points. Retention of surface-anchored mini-HA following SGF treatment was examined by confocal microscopy. As shown in Fig. 2A, preserved and uniform surface fluorescence was observed after SGF exposure, with no detectable antigen aggregation or loss. Consistently, flow cytometric analysis confirmed the stability of surface-displayed mini-HA (Fig. 2B). BCA assays revealed negligible protein release throughout the incubation period, indicating strong resistance of the displayed antigen to acidic and proteolytic degradation (Fig. 2C). Collectively, these results indicate that mini-HA remains stably anchored to the yeast surface under simulated gastric conditions.

### Surface anchoring of mini-HA is preserved following heat inactivation at 60 °C

**Fig. 3:**
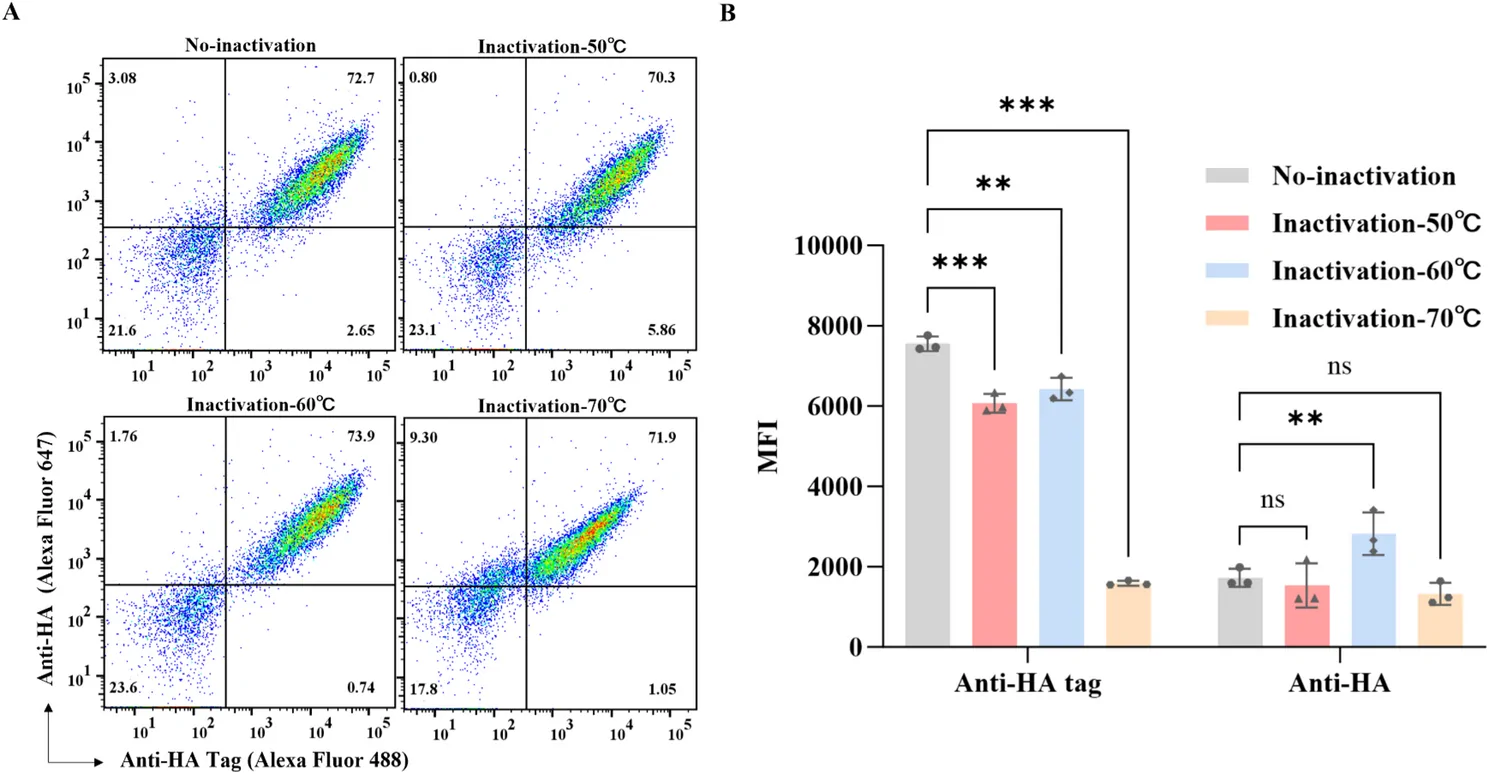
Thermal stability of YSD-mini-HA following heat inactivation. **A** Representative dual-label flow cytometric analysis of mini-HA and HA tag expression on yeast cells before and after heat inactivation. **B** MFI of mini-HA and HA tag before and after heat treatment. Data are presented as mean ± SE from three independent experiments. Statistical significance was determined by two-way ANOVA (**p < 0.01; ***p < 0.001; ns, not significant)

To prepare a non-replicative whole-yeast oral vaccine, YSD-mini-HA cells were subjected to heat inactivation. To confirm antigen retention after heat inactivation, dual-label flow cytometry was performed using antibodies against both the HA tag and mini-HA. A distinct double-positive population was observed both before and after heat treatment. The HA tag positive population decreased following heat treatment. Notably, mini-HA positivity after heat treatment at 60 °C was higher than that of the non-inactivated control and reached statistical significance (*p* < 0.05) (Fig. 3A, B). While the HA tag signal decreased, likely due to partial denaturation of the epitope tag, the mini‑HA conformational epitope remained intact or even became more accessible. As shown in Fig. S1B, no colony growth was observed following heat treatment at 60 °C and 70 °C, indicating complete loss of yeast cell viability. Further trypan blue staining and counting confirmed that the inactivation rate reached 96.36 ± 1.34% at 60 °C. These findings indicate that heat inactivation at 60 °C preserves the surface anchoring and antigenic integrity of mini-HA on yeast cells.

### Oral administration of heat-inactivated YSD-mini-HA induces robust humoral immune responses

**Fig. 4:**
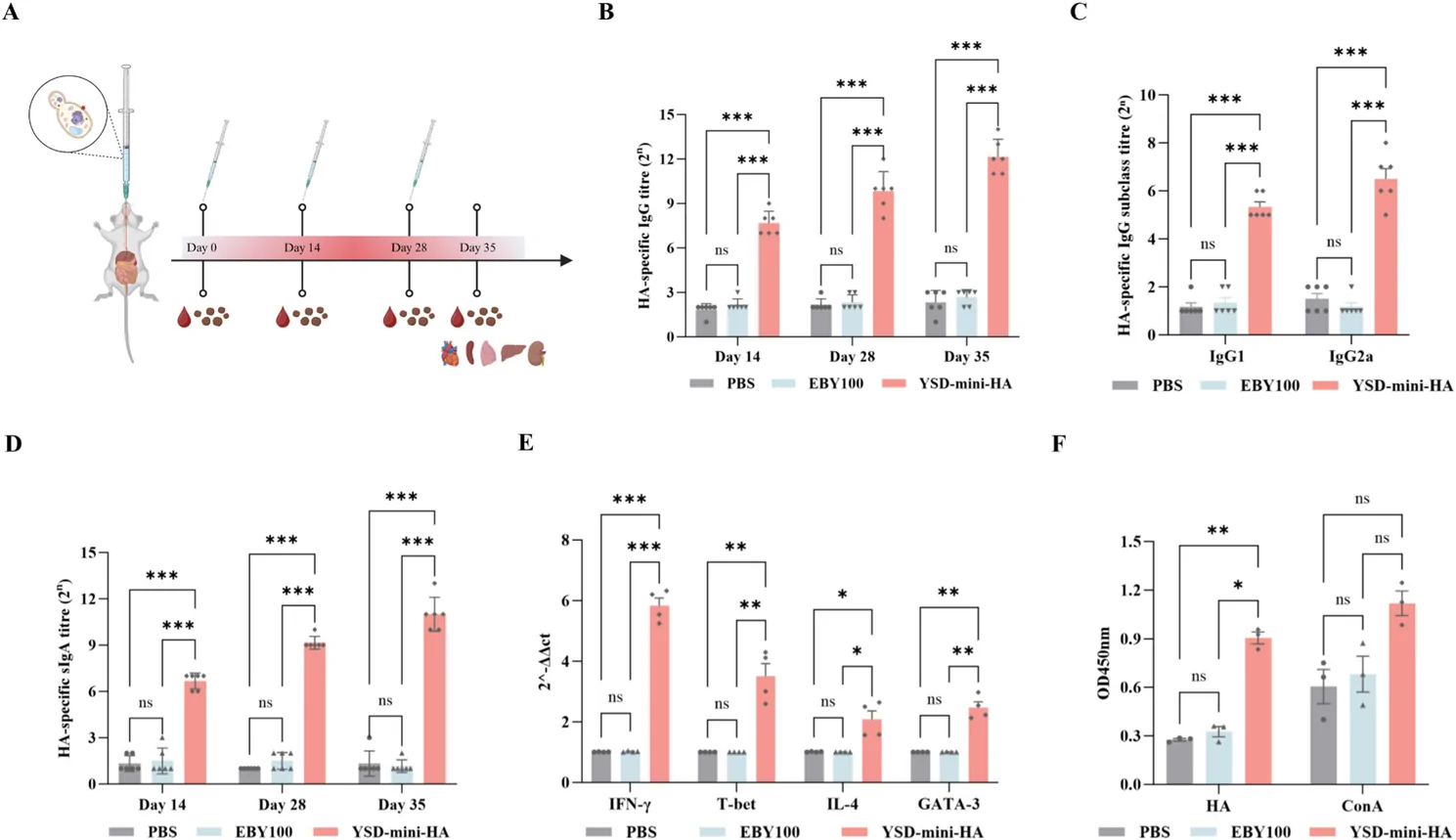
Humoral and cellular immune responses induced by oral YSD-mini-HA vaccination. **A** Schematic representation of the oral immunization schedule. **B** Serum HA-specific IgG titers measured on days 14, 28, and 35 after the initial immunization (*n* = 6/group). **C** IgG1 and IgG2a endpoint titers were determined in mouse sera at 35 days post-primary immunization (*n* = 6/group). **D** Fecal HA-specific sIgA titers measured at corresponding time points (*n* = 6/group). **E** RT-qPCR analysis of Th1- and Th2-associated cytokine mRNA expression levels in splenocytes (*n* = 4/group). **F** Proliferative responses of splenocytes following in vitro restimulation with HA antigen (*n* = 3/group). Data are shown as mean ± SE. Statistical significance was determined by two-way ANOVA (*p < 0.05; **p < 0.01; ***p < 0.001; ns, not significant)

The immunogenicity of the YSD-mini-HA oral vaccine was evaluated in BALB/c mice. Animals were randomly assigned to receive heat-inactivated YSD-mini-HA, heat-inactivated EBY100, or PBS by oral administration according to the immunization schedule shown in Fig. 4A. No HA-specific serum IgG antibodies were detected in mice receiving PBS or EBY100 at any time point.

In contrast, mice immunized with YSD-mini-HA exhibited a significant increase in HA-specific serum IgG levels as early as day 14 post-immunization (*p* < 0.001), with antibody titers increasing further following booster doses (Fig. 4B). After three immunizations, the geometric mean titer (GMT) of serum IgG reached 2^12.17^. To further define the T-helper cell response profile elicited by YSD-mini-HA, we measured serum titers of HA-specific IgG2a (Th1-associated) and IgG1 (Th2-associated) antibodies following the third immunization. As shown in Fig. 4C, the YSD-mini-HA-immunized group exhibited significantly elevated IgG2a and IgG1 antibody titers compared with both the PBS and EBY100 control groups (*p* < 0.001). Notably, the calculated IgG2a/IgG1 ratio in the YSD-mini-HA group was markedly greater than 1.0, indicative of a Th1-biased immune response.

Mucosal immune responses were assessed by measuring HA-specific secretory IgA (sIgA) levels in fecal samples. Robust sIgA responses were detected exclusively in the YSD-mini-HA-immunized group, with titers increasing over time and reaching a GMT of 2^11^ after the third immunization (*p* < 0.001) (Fig. 4D). These results demonstrate that oral administration of heat-stabilized YSD-mini-HA elicits strong systemic and mucosal humoral immune responses.

### YSD-mini-HA elicits antigen-specific cellular immune responses with a Th1-biased profile

Cellular immune responses induced by the oral vaccine were evaluated seven days after the final immunization. RT-qPCR analysis of splenic tissues revealed that mice immunized with YSD-mini-HA exhibited significantly increased expression of Th1-associated markers, including IFN-γ and T-bet, accompanied by moderate upregulation of Th2-associated cytokines (Fig. 4E). This cytokine profile indicates a mixed Th1/Th2 response with a pronounced Th1 bias.

Antigen-specific lymphocyte proliferation was assessed by CCK-8 assay following in vitro restimulation with HA protein. Splenocytes from YSD-mini-HA-immunized mice exhibited significantly enhanced proliferative responses compared with those from PBS- and EBY100-treated control groups (Fig. 4F). The proliferative response exceeded background levels and was antigen-specific, as no significant differences were observed between PBS and EBY100 controls. These results demonstrate that oral immunization with YSD-mini-HA induces robust antigen-specific cellular immune responses.

### Safety and biocompatibility of oral YSD-mini-HA vaccination

**Fig. 5:**
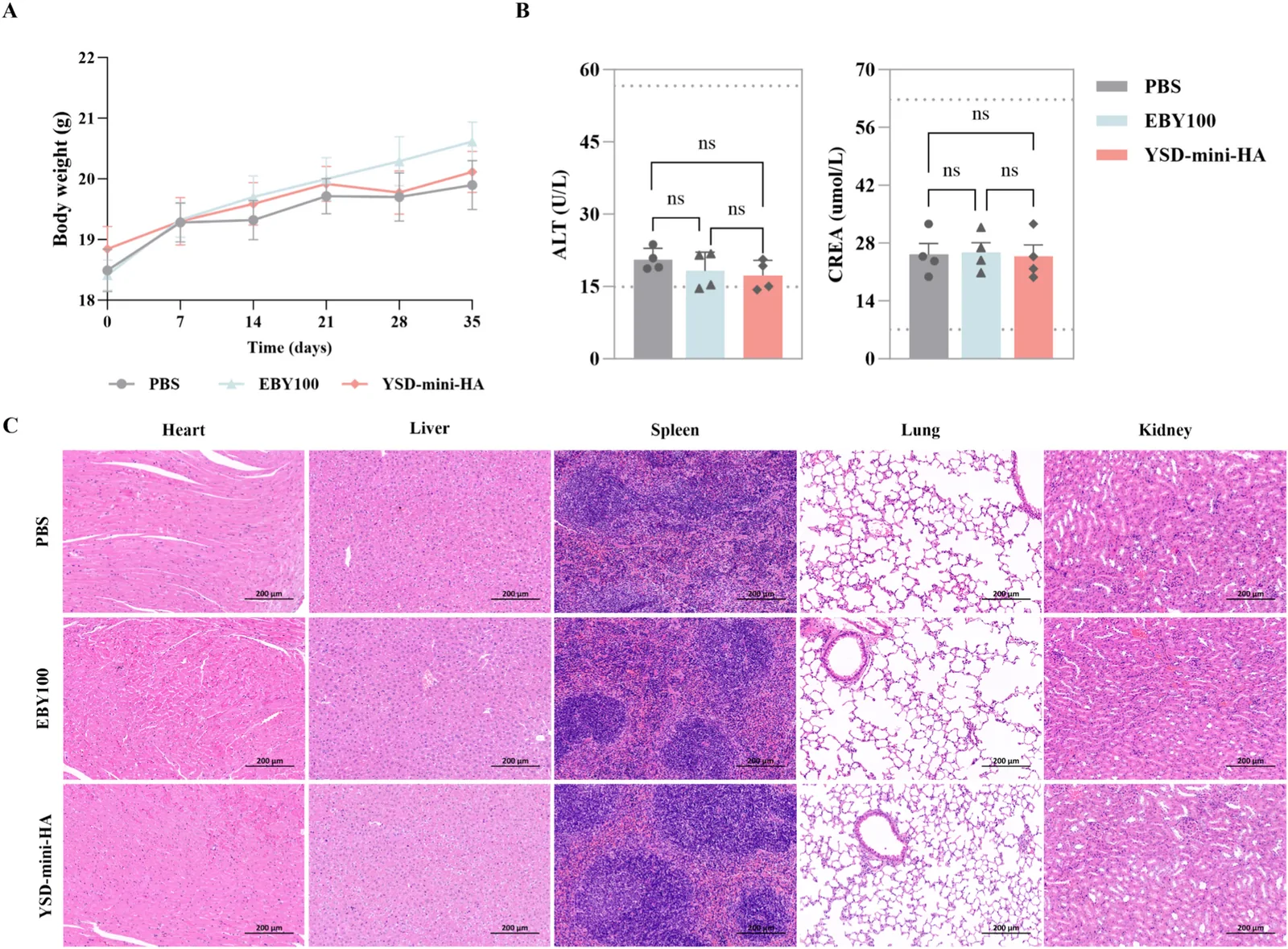
Safety and reactogenicity assessment of oral YSD-mini-HA vaccination. **A** Body weight changes in mice following oral immunization (*n* = 6/group). **B** Serum biochemical analysis of ALT and CREA levels after the final immunization (*n* = 3/group). **C** Representative H&E-stained sections of major organs (heart, liver, spleen, lung and kidney) collected after vaccination (*n* = 3/group). Scale bar, 200 µm. Statistical significance was determined by one-way ANOVA (ns, not significant).

The safety profile of the YSD-mini-HA oral vaccine was evaluated throughout the immunization period. No signs of distress, abnormal behavior, or mortality were observed in any group. Body weight increased steadily in all mice, with no significant differences between vaccinated and control groups (Fig. 5A).

Serum biochemical analyses revealed no significant differences in ALT or CREA levels between the YSD-mini-HA group and controls following three immunizations (Fig. 5B). Histopathological examination of the heart, liver, spleen, lung, kidney revealed no detectable pathological abnormalities in vaccinated mice compared with controls (Fig. 5C). Compared with parenteral vaccines, the safety assessment of oral vaccines requires particular attention to local mucosal tolerance. Consistent with this, H&E staining of gastrointestinal tissues from immunized mice revealed no evident pathological changes, including inflammatory cell infiltration, edema, or necrosis (Fig. S2). These findings collectively support that the YSD-mini-HA oral vaccine elicits effective mucosal immunity while maintaining excellent gastrointestinal tolerance and biosafety. Notably, mice immunized with YSD-mini-HA exhibited a modest but significant increase in spleen index (*p* < 0.05) (Table S2).

## Discussion

Yeast surface display has emerged as a versatile platform for heterologous protein expression and recombinant antigen presentation, with increasing applications in microbial engineering, biocatalysis, and vaccine delivery. In the present study, we engineered a *S. cerevisiae* surface display system for presentation of a rationally designed influenza mini-HA antigen and evaluated its stability and functional immunogenicity following oral administration. Seasonal influenza viruses continuously undergo antigenic drift and occasional antigenic shift, which can reduce the effectiveness of strain-matched vaccines (Chai et al. 2025; Zhang et al. 2024). Previous studies have demonstrated the feasibility of mini-HA immunogens using intranasal protein vaccines and bacterial oral delivery systems (van der Lubbe et al. 2018; Wang et al. 2025; Zhou et al. 2024). Our findings further support the application of yeast surface display as a stable and scalable system for recombinant antigen presentation and oral delivery.

Despite these advances, the development of oral vaccine systems that combine antigen stability, intrinsic adjuvant activity, biosafety, and scalability remains an active area of investigation. *S. cerevisiae* represents a well-established microbial platform for heterologous protein expression and oral antigen delivery, yet its application in surface-displayed oral mini-HA vaccines has been insufficiently explored. In the present study, we engineered a yeast surface display system in which a rationally designed mini-HA antigen was anchored to the yeast cell wall through the Aga1p-Aga2p anchoring mechanism. The mini-HA construct was designed to remove the hypervariable head domain while preserving conserved stem epitopes. In addition, regions implicated in molecular mimicry between HA-derived peptides and hypocretin-associated sequences were omitted to reduce potential safety concerns associated with narcolepsy-related autoimmunity (Buonocore and van der Most 2022; Ollila et al. 2023; Xu et al. 2024). This antigen engineering strategy was intended to improve the safety profile of the construct while maintaining conserved antigenic features.

The selection of *S. cerevisiae* as the expression and delivery platform was motivated by both biological and engineering considerations. Unlike prokaryotic expression platforms such as *Escherichia coli*, which lack machinery for post-translational glycosylation and frequently drive recombinant protein aggregation into insoluble inclusion bodies (Sandomenico et al. 2020), yeast supports N-linked glycosylation yet possess an inherently divergent glycan-processing programme compared with mammalian cells. Both systems initially generate the Man_8_GlcNAc_2_ precursor in the endoplasmic reticulum; however, upon entry into the Golgi apparatus, their pathways diverge markedly. Mammalian cells trim mannose residues and subsequently append N-acetylglucosamine, galactose, sialic acid and core fucose to generate complex- or hybrid-type N-glycans (Hossler et al. 2009), whereas yeast progressively elongates mannose chains to produce hypermannosylated, high-mannose structures (Burnap et al. 2025). As a type I transmembrane glycoprotein, HA contains conserved NXS/T motifs in which asparagine residues can undergo co‑translational or post‑translational N‑glycosylation (Huang et al. 2020). Consistent with this, Western blot analysis revealed that the mini‑HA protein displayed an apparent molecular weight higher than its theoretical prediction. Notably, treatment with peptide N‑glycosidase F (PNGase F) reduced the molecular weight to near the calculated value, confirming that the protein predominantly underwent N‑linked glycosylation during expression in yeast (Ge and Ross 2021).

This glycoform discrepancy exerts a dual effect on yeast-based oral vaccines. On one hand, abundant terminal mannose residues can be recognized by mannose receptors (including CD206 and DC-SIGN) on intestinal dendritic cells and M cells, facilitating receptor-mediated endocytosis and enhancing antigen uptake, thereby effectively eliciting mucosal secretory IgA (sIgA) responses (Baumann et al. 2010; Paurević et al. 2024). On the other hand, excessive mannose chains may sterically shield neutralizing epitopes, thereby attenuating the induction of neutralizing antibodies against native viral glycoproteins (Job et al. 2013; Kumar et al. 2025; Tate et al. 2014). To date, yeast-based vaccine platforms have shown promising results against multiple viral pathogens, including SARS-CoV-2 (Xing et al. 2022), H5N1 (Lei et al. 2016), and H7N9 (Lei et al. 2020). A key advantage of yeast lies in the β-glucans present in its cell wall, which function as intrinsic immunostimulatory molecules capable of enhancing antigen immunogenicity (Han et al. 2020; Kang et al. 2022). Previous studies have shown that orally administered yeast-based vaccines can accumulate in Peyer’s patches and mesenteric lymph nodes, thereby facilitating antigen presentation and mucosal immune priming (Chen et al. 2025).

Consistent with these properties, our data showed efficient surface expression of mini-HA on *S. cerevisiae* together with favorable physicochemical stability relevant to oral administration. The displayed antigen retained stable surface localization following exposure to simulated gastric fluid, indicating that a substantial proportion of surface-anchored antigen may remain intact during gastric transit. These observations are consistent with previous reports describing the stability of yeast surface-displayed antigens under simulated gastrointestinal conditions (Chen et al. 2025; Si et al. 2025). In addition, heat inactivation at 60 °C preserved mini-HA antigen display, in agreement with previous studies showing that moderate thermal treatment does not substantially compromise yeast surface-anchored proteins (Xing et al. 2022). This characteristic may provide practical advantages for biosafety, storage, and downstream manufacturing. Furthermore, heat-induced remodeling of the yeast cell wall has been reported to increase β-glucan exposure, which may further contribute to immunostimulatory activity (Bazan et al. 2014; Li et al. 2020; Ribeiro et al. 2022). From an engineering perspective, the combination of stable surface display and tolerance to acidic and thermal stress highlights the robustness of this yeast display platform for recombinant antigen presentation.

These favorable platform characteristics provided the basis for subsequent in vivo immunogenicity evaluation. Because influenza virus infection is initiated at mucosal surfaces, oral antigen delivery represents a rational strategy for inducing mucosal immunity. Oral administration of YSD-mini-HA induced antigen-specific systemic IgG responses together with robust mucosal sIgA production, indicating activation of both systemic and mucosal immune compartments. Although the magnitude of serum IgG responses was lower than that reported for highly immunogenic injectable vaccines (Wang et al. 2025), the induction of mucosal immunity may contribute to frontline defense at the site of viral entry, which is not typically achieved by conventional parenteral vaccines (van der Lubbe et al. 2018). Further studies will be required to evaluate neutralizing activity, durability of antibody responses, and the extent of cross-protection against antigenically diverse influenza strains.

In addition to humoral immunity, cellular immune responses are important for antiviral protection. The YSD-mini-HA vaccine elicited a mixed Th1/Th2 immune response with a marked Th1 bias, as characterized by significantly elevated serum titers of HA-specific IgG2a relative to IgG1, yielding an IgG2a/IgG1 ratio substantially greater than 1.0, together with upregulated splenic expression of IFN-γ and T-bet. This Th1-skewed profile is consistent with prior reports on yeast-based influenza and SARS-CoV-2 vaccine platforms (Lei et al. 2016; Si et al. 2025). Th1-associated responses contribute to macrophage activation and cytotoxic T-cell activity, whereas Th2-associated responses support B-cell activation and antibody production (Iwata et al. 2017). Antigen-specific lymphocyte proliferation further supported functional cellular immune activation. The observed Th1-biased response may be associated, at least in part, with the immunostimulatory properties of yeast-derived β-glucans, which can interact with pattern-recognition receptors and promote antigen presentation. However, the present study did not directly investigate the molecular mechanisms underlying T-cell polarization, and further studies will be required to define the relative contributions of CD4^+^ and CD8^+^ T-cell subsets as well as the specific role of yeast-derived adjuvanticity.

Several limitations should be acknowledged. First, viral challenge experiments were not performed in the present study, and therefore protective efficacy remains to be established. Second, the neutralizing activity of vaccine-induced antibodies was not evaluated. In addition, cytokine analyses were limited to transcriptional profiling rather than direct functional characterization of T-cell responses. Future studies will focus on evaluating cross-protective efficacy, defining cellular immune mechanisms, and assessing the durability of vaccine-induced immune memory.

## Conclusions

In conclusion, this study supports the feasibility of integrating a rationally engineered, safety-enhanced mini-HA antigen with a yeast surface display platform for oral antigen delivery. The YSD-mini-HA system-maintained antigen stability under simulated gastric conditions and following thermal inactivation while inducing coordinated mucosal and systemic immune responses in mice. Within the scope of this study, the platform was well tolerated, with no detectable adverse effects on body weight, serum biochemical markers, or tissue histopathology.

Collectively, these findings provide preliminary evidence for the use of yeast surface display as a potential platform for oral recombinant antigen presentation. The stability of the system under acidic and thermal stress conditions may offer practical advantages for manufacturing, storage, and large-scale application. Future studies will be needed to further evaluate cross-protective efficacy, cellular immune mechanisms, and the broader applicability of the platform for stable surface presentation of recombinant proteins in oral delivery systems.

## Supplementary Information

Below is the link to the electronic supplementary material.

Supplementary Material 1

## Data Availability

The datasets and materials supporting the findings of this study are available from the corresponding author upon reasonable request.
