# Supplementary material for: Stable surface display of a conserved influenza mini-HA antigen in Saccharomyces cerevisiae for oral delivery
Source: AMB Express. 2026 Aug 16;16:113. doi: 10.1186/s13568-026-02101-w (PMC13755293; doi:10.1186/s13568-026-02101-w)
Supplement: Supplementary file 1 [file 13568_2026_2101_MOESM1_ESM.docx]

**Supplementary Data S1**

**The sequence of the mini-HA construct was as follows:**

DTLCIGYHANNSTDTVDTVLEKNVTVTHSVNLLEDggggKYVCSTKLRLATGLRNKPSKQSQGLFGAIAGFTEGGWTGMVDGWYGYHHQNEQGSGYAADLKSTQNAIDKITNKVNSVIEKMNTQYTAIGCEYNHSEKCMKQIEDKIEEIESKDIWCYNAELLVLLENERTLDYHDSNVKNLYEKVRSQLKNNAKEIGNGCFEFYHKCDNTCMESVKNGTYDYPKYSEEAKLNREEIDGVKLESTRIYQILAenlyfqsGSGSGYIPEAPRDGQAYVRKDGEWVLLSTFLgsgs

**The DNA coding sequence of the construct was as follows:**

GATACACTCTGCATCGGTTATCATGCGAACAACAGCACGGATACGGTGGATACAGTACTAGAGAAGAACGTTACCGTCACTCACAGTGTTAATTTATTGGAAGATGGTGGAGGGGGTAAGTACGTTTGTTCTACCAAGTTGCGACTAGCGACGGGACTGCGAAACAAACCCTCGAAACAGTCGCAGGGATTATTCGGTGCTATTGCTGGTTTCACCGAAGGTGGTTGGACTGGTATGGTTGACGGCTGGTACGGTTATCATCACCAAAATGAACAAGGTTCGGGCTACGCTGCTGACTTGAAGTCTACACAGAATGCAATAGACAAAATCACCAACAAGGTCAACAGCGTGATCGAAAAGATGAACACTCAATACACTGCGATAGGCTGTGAATACAACCACTCTGAAAAGTGTATGAAGCAAATTGAAGACAAGATCGAGGAGATTGAATCCAAGGATATCTGGTGTTACAACGCGGAGCTACTAGTACTCCTAGAGAACGAAAGAACTTTGGACTACCACGATTCTAATGTGAAAAATCTATATGAGAAAGTACGATCGCAATTGAAAAATAACGCCAAGGAAATTGGTAACGGTTGTTTTGAATTTTATCACAAGTGCGACAACACCTGCATGGAATCTGTAAAAAATGGGACATACGACTACCCGAAATATTCTGAAGAGGCGAAATTGAACCGTGAGGAAATTGACGGGGTCAAATTGGAATCGACCAGAATCTACCAAATCTTAGCGGAGAATTTGTACTTCCAATCCGGATCAGGTTCTGGATATATTCCAGAAGCTCCTCGCGATGGCCAGGCCTACGTCAGAAAGGACGGTGAATGGGTTTTGTTAAGTACATTTCTGGGCAGCGGCTCG

**Figure S1**	Plate streaking results after inactivation at different temperatures.

(A) YSD-mini-HA cultures without inactivation were streaked on SD-Ura-Leu plates.

(B) YSD-mini-HA cultures after heat inactivation at the indicated temperatures were streaked on SD-Ura-Leu plates.

(C) Trypan blue staining and viability quantification of yeast cells after heat inactivation.

Statistical significance was determined by one-way ANOVA (*** *p* < 0.001; ns, not significant).

Experiments were repeated three times independently with similar results.

**Figure S2**	Representative H&E-stained sections of stomach and intestine collected from immunized mice (n=3/group). Scale bar, 200 µm.

**TABLE S1	Primers for RT-qPCR.**

| Gene name (Mice) | Forward primer sequence (5′-3′) | Reverse primer sequence (5′-3′) | Size (bp) |
| --- | --- | --- | --- |
| IFN-γ | AGACAATCAGGCCATCAGCA | TGGACCTGTGGGTTGTTGAC | 135 |
| IL-4 | CCATATCCACGGATGCGACA | AAGCACCTTGGAAGCCCTAC | 166 |
| T-bet | ATTGCCCGCGGGGTTG | GACAGGAATGGGAACATTCGC | 135 |
| GATA-3 | GGTCAAGGCAACCACGTC | CATCCAGCCAGGGCAGAG | 133 |
| β-actin | TACCCAGGCATTGCTGACAGG | ACTTGCGGTGCACGATGGA | 205 |

**TABLE S2	Systemic safety evaluation of heat-inactivated yeast displayed vaccine in immunized mice.**

| Group | Organ Index（mg/g） | | | | |
| --- | --- | --- | --- | --- | --- |
|  | Heart | Liver | Spleen | Lung | Kidney |
| YSD-mini-HA | 0.556 ± 0.019 | 5.021 ± 0.124 | 0.767 ± 0.040^*^ | 0.816 ± 0.017 | 1.384 ± 0.063 |
| EBY100 | 0.521 ± 0.005 | 4.465 ± 0.105 | 0.468 ± 0.016 | 0.728 ± 0.023 | 1.315 ± 0.011 |
| PBS | 0.562 ± 0.010 | 4.286 ± 0.193 | 0.435 ± 0.021 | 0.819 ± 0.0583 | 1.341 ± 0.024 |

Note: Data are shown as mean ± SE. An asterisk (*) denotes a statistically significant difference from the control group *(p* < 0.05*).*
